# Bleeding complications related to external ventricular drainage placement in patients with ruptured intracranial aneurysms: a single-center study

**DOI:** 10.3389/fsurg.2024.1403668

**Published:** 2024-10-15

**Authors:** Yue Tang, Xiangping Zhong, Tingting Lin, Fujun Zuo, Min Fu, Li Wang, Xiaodu Yu, Dong Liu, Jincan Zhang

**Affiliations:** ^1^ Department of Neurosurgery, The Fourth Hospital of Changsha, Changsha, China; ^2^ Department of Neurosurgery, The Central Hospital of Yongzhou, Yongzhou, China

**Keywords:** aneurysmal subarachnoid hemorrhage, external ventricular drainage, rebleeding, antiplatelet, hematoma

## Abstract

**Objective:**

Acute aneurysmal rupture can be treated with endovascular therapy or surgical clipping. For patients with concurrent acute hydrocephalus, the placement of an external ventricular drainage (EVD) is required. This study aims to investigate the impact of pre-treatment EVD placement on rebleeding in ruptured aneurysms and to examine the influence of dual antiplatelet therapy and the sequencing of dual antiplatelet therapy with EVD placement on EVD-related hematomas.

**Methods:**

We reviewed the clinical data of 83 patients with ruptured aneurysms who underwent EVD placement from a total of 606 aneurysm patients consecutively admitted between January 2018 and January 2023. The analysis focused on the impact of pre-treatment EVD placement on aneurysmal rebleeding and the effect of dual antiplatelet therapy and its sequencing with EVD placement on EVD-related hematomas.

**Results:**

Among the 503 patients with ruptured aneurysms, 83 required EVD placement. EVD was placed before aneurysm treatment in 63 patients and after treatment in 20 patients. The number of aneurysmal rebleeding cases in the pre-treatment EVD group and non-EVD group was 1 (1.6%) and 20 (4.8%), respectively (*p* = 0.406). 31 patients (37.3%) underwent stent-assisted embolization or flow diversion requiring dual antiplatelet therapy, while 52 patients (62.7%) underwent simple embolization or surgical clipping without antiplatelet therapy. EVD-related hematomas occurred in 14 patients (16.9%), with 10 cases (32.3%) in those receiving dual antiplatelet therapy and 4 cases (7.7%) in those not receiving antiplatelet therapy (*p* = 0.01). Among 16 patients who had EVD placed before dual antiplatelet therapy, 4 (25%) developed EVD-related hematomas. Of the 15 patients who had EVD placed after dual antiplatelet therapy, 6 (40%) developed EVD-related hematomas (*p* = 0.458).

**Conclusion:**

In patients with aneurysmal subarachnoid hemorrhage (aSAH) and acute hydrocephalus, the placement of EVD before aneurysm treatment does not increase the risk of rebleeding. However, dual antiplatelet therapy increases the risk of EVD-related hematoma, and the sequence of EVD placement relative to dual antiplatelet therapy does not appear to significantly affect the outcome of EVD-related hematoma.

## Background

External ventricular drainage (EVD) is one of the most crucial and common emergency procedures in neurosurgery, primarily utilized for the restoration or monitoring of intracranial pressure (ICP). Acute hydrocephalus is a frequent complication of aneurysmal subarachnoid hemorrhage (aSAH), and EVD can facilitate the rapid normalization of elevated ICP, rendering patients more suitable for surgical intervention and improving their clinical status (1–3). The timing of EVD placement in relation to aneurysm treatment remains controversial, as pre-treatment placement may induce rebleeding and post-treatment placement may delay the normalization of elevated ICP (2, 4–5). With the advancement of neurointerventional materials and techniques, endovascular treatment (EVT) has become a predominant trend for ruptured aneurysm management (6, 7). In patients requiring stent implantation, dual antiplatelet therapy is necessary to prevent stent-related thromboembolic complications (8, 9). Given that EVD carries a risk of hemorrhage (10–13), dual antiplatelet therapy could theoretically increase the risk of EVD-related hematoma. Furthermore, the impact of the sequence of dual antiplatelet therapy and EVD placement on EVD-related hematoma is not well understood. The purpose of this study is to report on our experience with EVD during the treatment of ruptured aneurysms, in order to provide a reference for the selection of clinical treatment protocols.

## Study population

**Figure 1:**
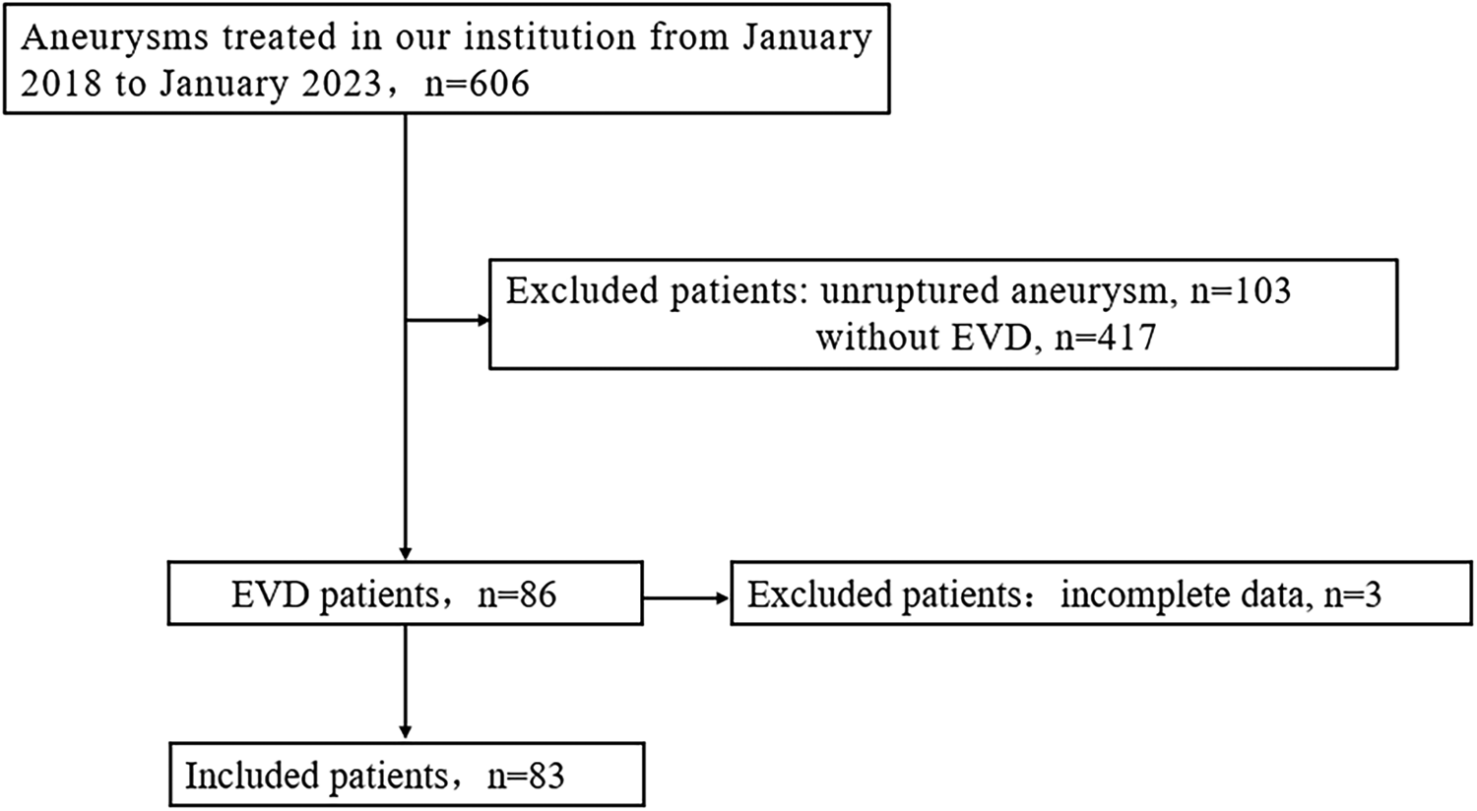
Flowchart of patient selection. EVD, external ventricular drainage.

We retrospectively analyzed the clinical data of 606 consecutive adult aneurysm patients treated from January 2018 to January 2023. Inclusion criteria were: (1) confirmed aneurysmal subarachnoid hemorrhage (aSAH) through imaging prior to surgery, (2) clinical and radiological confirmation of acute hydrocephalus warranting ventricular drainage, and (3) complete clinical data; ultimately, 83 patients were included in the study (Figure 1). EVD-related hematomas were defined as any hematoma in the perioperative drainage tube area. A symptomatic hematoma was defined as one requiring further surgical intervention or causing a persistent deterioration in daily neurological function. Aneurysmal rebleeding was defined as a sudden clinical deterioration accompanied by an increase in subarachnoid, intracerebral, or intraventricular hematoma confirmed by CT scan. This retrospective review received approval from the hospital's ethics committee, and informed consent from patients was deemed unnecessary.

## Treatment strategy

Patients diagnosed with aSAH were admitted to the neuro-intensive care unit and administered analgesics, sedatives, antihypertensives, and antifibrinolytic therapy. A strict protocol was maintained to keep systolic blood pressure (SBP) between 120 and 140 mmHg until the ruptured aneurysm was secured. Patients with unstable respiration were intubated and managed with respiratory support. Close monitoring of consciousness, pupil changes, vital signs, and dynamic repeat head imaging were conducted.

## Antiplatelet therapy

The treatment modality for aneurysms was recommended after departmental discussion and ultimately decided by the family. Patients expected to require stent placement were administered aspirin 300 mg and clopidogrel 300 mg orally or via nasogastric tube 6 h before surgery, with intraoperative heparinization at 60 u/kg. During stent insertion, tirofiban (5 mg/100 ml) at 4–6 ml was intravenously administered over 5 min as per the situation; postoperatively, clopidogrel 75 mg and aspirin 100 mg were given daily until a 3-month postoperative review.

## Lateral ventriculostomy

For patients with acute hydrocephalus, EVD is performed bedside or in the operating room. The 10F or 12F lateral ventricular drainage tube is inserted through Kocher's point at a depth of 5–7 cm and placed into the frontal horn of the left or right lateral ventricle. After successful puncture, a small amount of cerebrospinal fluid is slowly released.

## Statistical analysis

Statistical analysis of clinical data among the groups was performed using SPSS 26.0. Continuous variables were expressed as mean ± standard deviation, and categorical variables were presented as number-percentage. Independent sample *T*-tests/univariate ANOVA, Fisher's exact test, and chi-square tests were utilized for analysis. A *P*-value < 0.05 was considered statistically significant.

## Results

### EVD-Related aneurysmal rebleeding

Among the 606 aneurysm patients treated, there were 103 cases of unruptured aneurysms and 503 cases of ruptured aneurysms. Of these, EVD was not placed in 417 patients, while 86 patients with ruptured aneurysms had EVD placement, with 63 cases before aneurysm treatment and 23 cases after treatment (3 patients were excluded from this study for not having a follow-up CT). The clinical data for the pre-treatment EVD group and the non-EVD group is detailed in Table 1; there were no statistical differences between the two groups in terms of age, gender ratio, aneurysm location, and treatment modality. Compared to the non-EVD group, the pre-treatment EVD group had a smaller average aneurysm diameter (4.7 ± 2.0 vs. 5.5 ± 2.6, *P* < 0.001), higher initial Hunt-Hess scores (3.8 ± 0.9 vs. 2.5 ± 1.1, *P* < 0.001), and higher Fisher scores (3.1 ± 0.8 vs. 2.3 ± 1.0, *P* < 0.001).

**Table 1:** Baseline characteristics of EVD and non EVD before treatment in patients with aSAH.

Characteristics	EVD group	Non EVD group	*P*
No. of patients	63	417	
Age, *y*			0.407
Mean ± SD	58.8 ± 10.7	60.3 ± 9.8	
Sex, *N*(%)			0.948
Male	20 (31.7%)	131 (31.4%)	
Female	43 (68.3%)	286 (68.6%)	
No. of aneurysms	67	449	
Aneurysm location, *N*(%)			0.464
Anterior circulation	52 (77.8%)	363 (80.8%)	
Posterior circulation	15 (22.2%)	86 (19.2%)	
Aneurysm diameter, *N*(%)			<0.001
<5 mm	33 (49.3%)	149 (33.2%)	
5–15 mm	34 (50.7%)	287 (63.9%)	
>15 mm	0 (0%)	13 (2.9%)	
Treatment method, *N*(%)			0.369
Endovascular treatment	19 (30.2%)	149 (35.7%)	
Surgical clipping	44 (69.8%)	268 (64.3%)	
Hunt-Hess score, *N*(%)			<0.001
I	0 (0%)	73 (17.5%)	
II	5 (7.9%)	153 (36.7%)	
III	19 (30.2%)	116 (27.8%)	
IV	22 (34.9%)	46 (11.0%)	
V	17 (27.0%)	29 (7.0%)	
Fisher score, *N*(%)			<0.001
1	1 (1.6%)	113 (27.1%)	
2	13 (20.6%)	135 (32.4%)	
3	27 (42.9%)	108 (25.9%)	
4	22 (34.9%)	61 (14.6%)	

EVD, External Ventricular Drainage; aSAH, Aneurysmal Subarachnoid Hemorrhage.

The pre-treatment EVD group had one case of rebleeding, which was treated with emergency craniotomy and aneurysm clipping, while the non-EVD group had 20 cases of rebleeding, all treated with emergency craniotomy and aneurysm clipping. The incidence of rebleeding were 1.6% (1/63) and 4.8% (20/417) respectively, *P* = 0.406, showing no statistical difference (Table 2).

**Table 2:** Comparison of the incidence of aneurysms rebleeding between EVD group and non EVD group.

Groups	*n*	Number with aneurysms rebleeding	Number without aneurysms rebleeding
EVD group	63	1	62
Non EVD group	417	20	397
*X* ^2^			0.689
*P*			0.406

*X*^2^, Chi-square test continuity correction.

### Relationship between dual antiplatelet therapy and EVD-related hematomas

Among the 83 EVD patients, none were on antiplatelet medication before the onset of the disease. Thirty-one patients received dual antiplatelet therapy, while 52 did not. Clinical data for both groups are detailed in Table 3, showing no statistical differences in age, gender ratio, and time from EVD to onset. Compared to the non-antiplatelet group, the dual antiplatelet group had a higher proportion of posterior circulation aneurysms (38.9% vs. 12.3%, *P* = 0.002), larger average aneurysm diameter (6.4 ± 2.9 vs. 4.2 ± 1.9, *P* < 0.001), and a higher proportion of endovascular treatment (100% vs. 15.4%, *P* < 0.001). The initial Hunt-Hess score was lower (3.5 ± 0.9 vs. 3.7 ± 1.0, *P* < 0.001), and the Fisher score was also lower (2.8 ± 0.8 vs. 3.1 ± 0.8, *P* = 0.046).

**Table 3:** Baseline characteristics of dual antiplatelet and non antiplatelet in patients of EVD with aSAH.

Characteristics	Dual Antiplatelet group	Non antiplatelet group	*P*
No. of patients	31	52	
Age, *y*			0.116
Mean ± SD	57.1 ± 10.3	59.5 ± 11.1	
Sex, *N*(%)			0.075
Male	15 (48.4%)	15 (28.8%)	
Female	16 (51.6%)	37 (71.2%)	
No. of aneurysms	36	57	
Aneurysm location, *N*(%)			0.002
Anterior circulation	22 (61.1%)	50 (87.7%)	
Posterior circulation	14 (38.9%)	7 (12.3%)	
Aneurysm diameter, *N*(%)			<0.001
<5 mm	11 (30.6%)	32 (56.1%)	
5–15 mm	25 (69.4%)	25 (43.9%)	
>15 mm	0 (0%)	0 (0%)	
Treatment method, *N*(%)			<0.001
Endovascular treatment	31 (100%)	8 (15.4%)	
Surgical clipping	0 (0%)	44 (84.6%)	
Hunt-Hess score, *N*(%)			<0.001
II	4 (12.9%)	7 (13.6%)	
III	10 (32.3%)	15 (28.8%)	
IV	13 (41.9%)	15 (28.8%)	
V	4 (12.9%)	15 (28.8%)	
Fisher score, *N*(%)			0.046
1	0 (0%)	1 (1.9%)	
2	14 (45.2%)	9 (17.3%)	
3	9 (29.0%)	24 (46.2%)	
4	8 (25.8%)	18 (34.6%)	
EVD sequence, *N*(%)			<0.001
Pre-treatment	16 (51.6%)	47 (90.4%)	
Post-treatment	15 (48.4%)	5 (9.6%)	
Onset time to EVD, h			0.511
Mean ± SD	45 ± 38.3	31.3 ± 35.4	
Hematoma volume, ml			0.47
Mean ± SD	8.3 ± 12.0	4.0 ± 2.2	

EVD, External Ventricular Drainage; aSAH, Aneurysmal Subarachnoid Hemorrhage.

Except for one patient who underwent bilateral frontal horn lateral ventricular drainage (in the dual antiplatelet group), all others had right frontal horn lateral ventricular drainage. Ten cases of EVD-related hematomas occurred in the dual antiplatelet group, and four in the non-antiplatelet group, with hematoma incidence of 32.3% (10/31) and 7.7% (4/52) respectively, *P* = 0.01, indicating a statistically significant difference (Table 4). The average volume of hematoma in the dual antiplatelet group was 8.3 ± 12 ml, compared to 4.0 ± 2.2 ml in the non-antiplatelet group, *P* = 0.47. One case progressed to symptomatic intracranial hematoma and underwent craniotomy for hematoma evacuation (dual antiplatelet group, 42 ml), while the remaining 13 cases were non-symptomatic hematomas and were treated conservatively.

**Table 4:** Comparison of the incidence of EVD-related hematoma between Dual antiplatelet group and non antiplatelet group.

Groups	*n*	Number with EVD-related hematoma	Number without EVD-related hematoma
Dual antiplatelet group	31	10	21
Non antiplatelet group	52	4	48
*X* ^2^			6.698
*P*			0.01

*X*^2^, Chi-square test continuity correction.

### Impact of EVD placement sequence with dual antiplatelet therapy on EVD-related hematomas

Among the 31 patients who underwent dual antiplatelet therapy, EVD was placed before dual antiplatelet therapy in 16 cases and after in 15 cases. Clinical data for both groups are detailed in Table 5. There were no statistical differences between the two groups in terms of age, gender ratio, aneurysm size, and initial Hunt-Hess and Fisher scores. Compared to the post-dual antiplatelet therapy EVD group, the pre-dual antiplatelet therapy EVD group had a higher proportion of posterior circulation (68.7% vs. 15.0%, *P* = 0.001) and a shorter duration from EVD to onset (19.2 ± 19.4 h vs. 72.6 ± 33.9 h, *P* < 0.001). In the pre-dual antiplatelet therapy EVD group, there were 2 initial CT hematomas, 2 delayed hematomas, totaling 4 patients with perioperative EVD-related hematomas, all of which were non-symptomatic. In the post-dual antiplatelet therapy EVD group, there were 4 initial CT hematomas, 2 delayed hematomas, totaling 6 patients with perioperative EVD-related hematomas, including 1 case that progressed to symptomatic hemorrhage (treated with craniotomy), the other 5 cases were non-symptomatic. The volume of hematomas in the pre- and post-dual antiplatelet therapy EVD groups were 5.3 ± 2.5 ml and 10.3 ± 14.2 ml respectively, *P* = 0.467. The incidence of initial CT hematomas were 12.5% (2/16) and 26.7% (4/15), *P* = 0.394 respectively, with no statistical difference, and the perioperative hematoma incidence was 25.0% (4/16) and 40.0% (6/15), *P* = 0.458, also with no statistical difference (Table 6).

**Table 5:** Baseline Characteristics of Dual antiplatelet in patients of EVD with aSAH.

Characteristics	Pre-Dual antiplatelet group	Post-Dual antiplatelet group	*P*
No. of patients	16	15	
Age, y			0.223
Mean ± SD	55.7 ± 7.3	58.9 ± 13.0	
Sex, *N*(%)			0.224
Male	6 (37.5%)	9 (60.0%)	
Female	10 (62.5%)	6 (40.0%)	
No. of aneurysms	16	20	
Aneurysm location, *N*(%)			0.001
Anterior circulation	5 (31.3%)	17 (85.0%)	
Posterior circulation	11 (68.7%)	3 (15.0%)	
Aneurysm diameter, *N*(%)			0.09
<5 mm	5 (31.3%)	6 (30.0%)	
5–15 mm	11 (68.7%)	14 (70.0%)	
Hunt-Hess score, *N*(%)			0.137
II	0 (0%)	4 (26.7%)	
III	7 (43.8%)	3 (20.0%)	
IV	7 (43.8%)	6 (40.0%)	
V	2 (12.4%)	2 (13.3%)	
Fisher score, *N* (%)			0.965
2	7 (43.8%)	7 (46.6%)	
3	5 (31.2%)	4 (26.7%)	
4	4 (25.0%)	4 (26.7%)	
Onset time to EVD, h			<0.001
Mean ± SD	19.2 ± 19.4	72.6 ± 33.9	

EVD, External Ventricular Drainage; aSAH, Aneurysmal Subarachnoid Hemorrhage; EVT, Endovascular Treatment.

**Table 6:** Comparison of the incidence of EVD-related hematoma between Pre-Dual antiplatelet group and Post-Dual antiplatelet group

Groups	*N*	EVD-related hematoma		*P*
		Yes	No	
Initial CT hematoma				
Pre-Dual antiplatelet group	16	2	14	0.394
Post-Dual antiplatelet group	15	4	11	
Perioperative hematoma				
Pre-Dual antiplatelet group	16	4	12	0.458
Post-Dual antiplatelet group	15	6	9	

## Discussion

### Analysis of EVD-related rebleeding

In patients with aneurysmal subarachnoid hemorrhage (aSAH) complicated by acute hydrocephalus, EVD can normalize elevated ICP and reduce the risk of secondary brain injury due to intracranial hypertension. Early placement of EVD seems to be a quite rational treatment choice (14, 15). However, there is concern that placement of EVD before treatment of the ruptured aneurysm may increase the risk of rebleeding (16, 17). In this context, our attempt to evaluate the safety of pre-treatment EVD placement found that among the 417 non-EVD patients with ruptured aneurysms, rebleeding occurred in 20 cases (4.8%), which is similar to figures reported in the literature. In the 63 patients with EVD placement, only 1 case (1.6%) experienced rebleeding, a lower rate than previously reported (18). The average duration from onset to EVD placement in the study reported was 16 h, whereas in our group it was 21.3 h. The main reason for rebleeding after EVD placement is the disruption of transmural pressure balance across the aneurysm after CSF drainage. It is still unclear whether there is a close relationship between the time from EVD insertion to the onset and rebleeding. Our data indicates that for patients with aSAH and concurrent acute hydrocephalus, pre-treatment EVD does not increase the risk of rebleeding and is a safe treatment choice.

### Analysis of EVD-related hematomas

Endovascular treatment is a safe and effective technique for the management of intracranial aneurysms (19–22). For patients requiring stent placement, perioperative dual antiplatelet medication is a necessary procedure (8, 9). Studies have shown that the combination of endovascular treatment with EVD can effectively control intracranial hypertension and improve the prognosis of patients with aSAH (23). A major concern is that dual antiplatelet therapy may increase the risk of EVD-related hemorrhage complications. In this context, we aimed to assess the impact of dual antiplatelet therapy, the placement of EVD, and the sequence of dual antiplatelet therapy on EVD hemorrhage complications. In our study, of the 83 EVD patients, 14 experienced EVD-related hematomas. The dual antiplatelet group had 10 cases of EVD-related hematomas, while the non-antiplatelet group had 4 cases, with a significantly higher incidence of EVD-related hematomas in the dual antiplatelet group (32.3%) compared to the non-antiplatelet group (7.7%). Dual antiplatelet treatment increased the risk of EVD-related hematoma, but there was no significant difference in the volume of hematomas between the two groups. When hemorrhage was detected on radiological examination, clopidogrel was discontinued in the context of dual antiplatelet therapy, while aspirin was continued, and head imaging was repeated. If the hematoma continued to expand, all antiplatelet medications were stopped. Only in the dual antiplatelet group was a hematoma observed to progress to symptomatic hemorrhage, which necessitated craniotomy for hematoma evacuation. The rest were non-symptomatic. This finding is consistent with previous studies, which suggest that most EVD-related hematomas associated with EVT are not clinically significant (24–26). Theoretically, the risk of EVD-related hematoma could increase if EVD is placed after antiplatelet therapy, making pre-antiplatelet therapy placement seemingly safer. In our study, we analyzed the impact of the sequence of EVD placement and dual antiplatelet therapy on EVD-related hematomas. In our data, the average time of EVD placement before dual antiplatelet therapy was 10.4 h, while it was 33.9 h after dual antiplatelet therapy. Although initial CT scans showed a higher tendency for hemorrhage when EVD was placed after dual antiplatelet therapy, there was no statistical difference, and neither was there a difference in the incidence of perioperative hematomas. However, EVD placement after dual antiplatelet therapy seemed to result in more severe hemorrhage. Although our study did not detect any statistically significant relationship between the sequence of EVD placement and the initiation of dual antiplatelet therapy in terms of impact on the risk of EVD related hematoma, it may be underpowered due to our limited sample size. Further research is needed to determine if placing an EVD before securing an aneurysm or initiating an antiplatelet agent results in a lower overall risk of bleeding complications.

Nevertheless, this study still has some limitations: (1) It is a single-center retrospective study that may be subject to selection bias affecting the outcomes; (2) The study has a relatively small sample size, and larger-scale, well-designed prospective randomized clinical trials are needed to determine the details of EVD use in the treatment of ruptured aneurysms.

## Conclusion

For patients with aSAH and concurrent acute hydrocephalus, the placement of EVD before aneurysm treatment does not increase the risk of rebleeding. Dual antiplatelet therapy increases the risk of EVD-related hematoma, but the sequence of EVD placement and antiplatelet therapy does not appear to significantly affect the outcome of EVD-related hematoma.

## Data Availability

The raw data supporting the conclusions of this article will be made available by the authors, without undue reservation.
